# A Practical Method to Prevent Cross-Infection When Performing Dermoscopy

**DOI:** 10.5826/dpc.1101a110

**Published:** 2021-01-29

**Authors:** Mohammed I. AlJasser

**Affiliations:** 1 Division of Dermatology, King Saud bin Abdulaziz University for Health Sciences, Riyadh, Saudi Arabia; 2 King Abdulla International Medical Research Center, Riyadh, Saudi Arabia

**Keywords:** pearl, dermoscopy, dermatoscopy, plastic wrap, rubber band, tie, wire, cross-infection, COVID-19

Dermoscopy is a valuable noninvasive diagnostic tool in dermatology. Contact dermoscopy is commonly used. Due to the potential risk of cross-infection by dermoscopy, several methods have been described to decrease this risk [1]. Commercially available disposable plastic covers are excellent for this purpose. However, cost and availability are limiting factors. Plastic wrap has been shown to be efficient in preventing transmission of viruses [2]. One disadvantage of this method is the limited flexibility of use. This is especially true when examining multiple body sites where the plastic sheet has to be moved to the next body site.

This issue can be resolved by using a small piece of plastic wrap and a rubber band (Figure 1). The plastic wrap is firmly stretched over the dermatoscope faceplate then fixed in place with the rubber band (Figure 2). Alternatively, a metal cable tie can be used (Figure 3). After completing the examination, this customized cover can be easily removed and discarded.

## Figures and Tables

**Figure 1:**
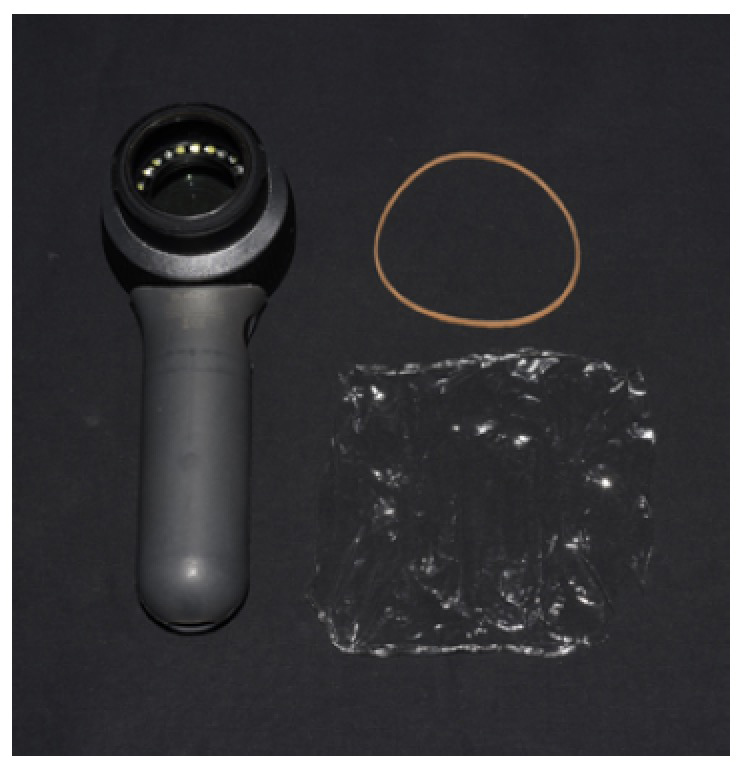
A small piece of plastic wrap and rubber band is required to create a custom dermoscopy faceplate cover.

**Figure 2:**
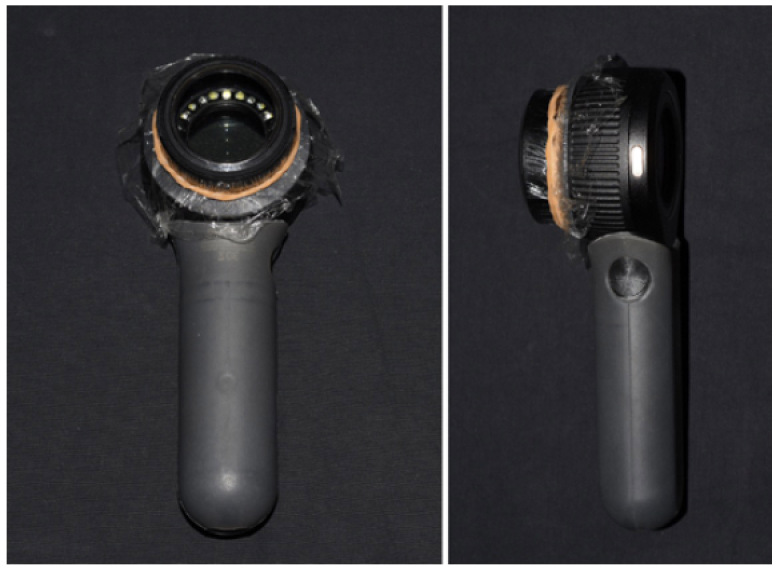
The plastic wrap is firmly stretched over the dermatoscope faceplate then fixed in place with the rubber band.

**Figure 3:**
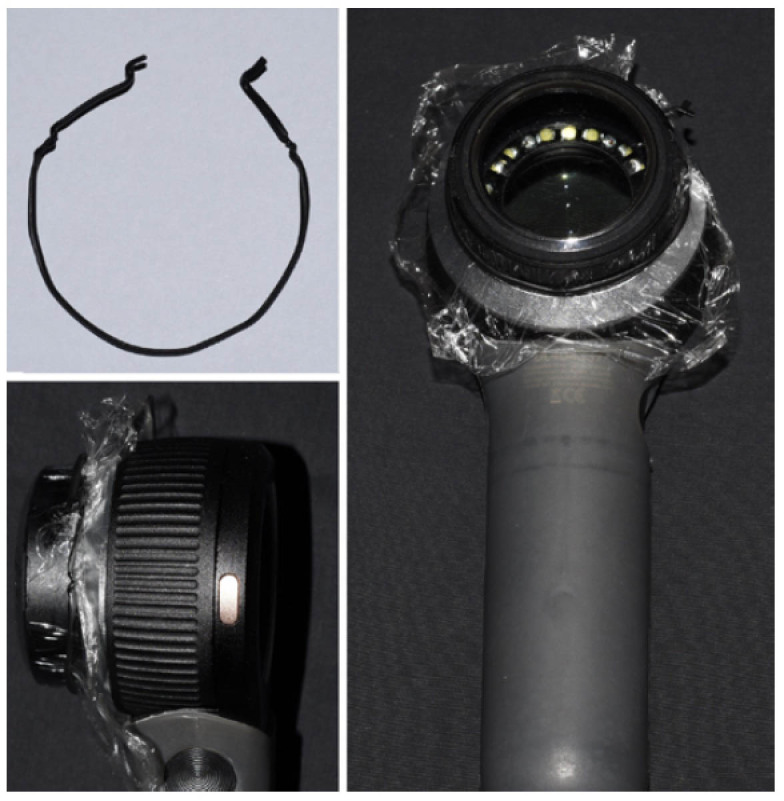
The plastic wrap can also be fixed in place using a metal cable tie.
